# Clinical Evaluation of Human Papillomavirus Screening With p16/Ki-67 Dual Stain Triage in a Large Organized Cervical Cancer Screening Program

**DOI:** 10.1001/jamainternmed.2019.0306

**Published:** 2019-05-13

**Authors:** Nicolas Wentzensen, Megan A. Clarke, Renee Bremer, Nancy Poitras, Diane Tokugawa, Patricia E. Goldhoff, Philip E. Castle, Mark Schiffman, Julie D. Kingery, Kiranjit K. Grewal, Alex Locke, Walter Kinney, Thomas S. Lorey

**Affiliations:** 1 Division of Cancer Epidemiology and Genetics, National Cancer Institute, National Institutes of Health, Rockville, Maryland; 2 Kaiser Permanente The Permanente Medical Group Regional Laboratory, Berkeley, California; 3 Department of Epidemiology and Population Health, Albert Einstein College of Medicine, Bronx, New York; 4 Global Coalition Against Cervical Cancer, Arlington, Virginia

## Abstract

**Importance:**

As cervical cancer screening transitions from Papanicolaou cytologic screening to primary human papillomavirus (HPV) testing worldwide, effective triage tests are needed to decide who among the HPV-positive women should receive further diagnostic evaluation to avoid unnecessary colposcopies and biopsies.

**Objective:**

To evaluate the performance of the p16/Ki-67 dual stain (DS) and HPV16/18 genotyping for the triage of HPV-positive women.

**Design, Setting, and Participants:**

A prospective observational study was conducted within the cervical cancer screening program at Kaiser Permanente Northern California of 3225 HPV-positive women undergoing HPV and Papanicolaou cytologic testing with a valid DS result from September 16 to October 31, 2015, with follow-up through December 31, 2018.

**Exposures:**

Human papillomavirus screening with partial genotyping and cytologic triage compared with DS triage.

**Main Outcomes and Measures:**

Cervical intraepithelial neoplasia grade 3 or more severe (CIN3+) and grade 2 or more severe (CIN2+), diagnosed within 3 years after sample collection.

**Results:**

A total of 3225 women (mean [SD] age, 37.9 [11.3] years) participated in the study. For triage of HPV-positive women with partial genotyping, DS showed better risk stratification for CIN3+ than did Papanicolaou cytologic testing, with women with positive DS results having a higher risk than women with positive Papanicolaou test results for CIN3+ (218 of 1818 [12.0%; 95% CI, 10.5%-13.5%] vs 219 of 2128 [10.3%; 95% CI, 9.0%-11.6%]; *P* = .005). Similarly, DS showed better risk stratification for CIN3+ compared with Papanicolaou cytologic testing in HPV-positive women, irrespective of genotyping. The greatest reassurance against CIN3+ was observed in HPV16/18-negative women with negative DS results, with a risk low enough to extend retesting intervals. Dual stain triage strategies required substantially fewer colposcopies per detection of CIN3+ compared with Papanicolaou cytologic testing, with a 32.1% (859 of 2677) reduction of colposcopies compared with the currently recommended triage strategy of HPV screening with Papanicolaou cytologic testing. Results for CIN2+ were very similar.

**Conclusions and Relevance:**

Triage of HPV-positive women with DS was superior to Papanicolaou cytologic testing in this study, demonstrating equal immediate detection of precancerous lesions and substantially reduced referral to colposcopy. These findings suggest that DS can safely replace Papanicolaou cytologic testing as a triage strategy for primary HPV screening, and that retesting intervals in HPV16/18-negative women with negative DS results can be safely extended to 3 years.

## Key Points

**Question:**

What are efficient approaches for triage of human papillomavirus–positive women in cervical cancer screening?

**Findings:**

This cohort study of 3225 women found that p16/Ki-67 dual stain, alone or in combination with human papillomavirus 16/18 genotyping, provides better risk stratification than comparable cytologic-based strategies.

**Meaning:**

Triage of human papillomavirus–positive women with dual stain may lead to lower referral to undergo colposcopy with similar detection of precancerous lesions compared with cytologic screening, making cervical cancer screening more efficient.

## Teaser

This cohort study evaluates the performance of the p16/Ki-67 dual stain and human papillomavirus (HPV) 16/18 genotyping for the triage of women with HPV infection.

## Introduction

Cervical cancer screening is shifting from primary Papanicolaou cytologic testing to primary human papillomavirus (HPV) testing worldwide.^1^ Women with negative results for HPV are reassured of a low risk of cervical cancer for many years and screening intervals can be safely extended compared with primary cytologic screening.^2^ Although HPV infections are common in the population, most HPV-positive women have transient infections. Three primary screening strategies are currently approved in the United States: Papanicolaou cytologic testing, HPV testing with Papanicolaou cytologic cotesting, and HPV testing with partial genotyping.^3^ For all screening approaches, triage tests are needed to decide which patients should be referred to undergo colposcopy for diagnostic evaluation.^4,5^ Per current HPV screening recommendations, HPV16/18-positive women are referred to undergo colposcopy, while women with positive results for other high-risk types of HPV undergo cytologic testing.^6,7^ Partial genotyping for HPV16 and HPV18 identifies the 2 genotypes with the highest risk of cancer,^8^ but cannot distinguish a transient infection from a prevalent precancerous lesion. Triage with Papanicolaou cytologic testing is subjective, and its sensitivity varies widely, requiring retesting of HPV-positive women with negative cytologic results.^9^

Primary screening and triage strategies should be evaluated together, because the safety and efficiency of a screening approach and subsequent management depends on the combination of component test results. An ideal screening and triage approach should identify as many precancerous lesions as possible, while referring as few women as possible to undergo colposcopy. Detection of p16/Ki-67 (dual stain [DS]) in cytologic specimens is an accurate marker for cervical precancerous lesions.^10,11,12,13,14,15^

We conducted a large clinical evaluation study at Kaiser Permanente Northern California (KPNC) evaluating DS together with HPV16/18 genotyping compared with the approved primary HPV screening strategy. We studied the performance of DS cytologic testing for triage of HPV-positive women, focusing on tradeoffs between colposcopy referral and detection of precancerous lesions in clinical practice.

## Methods

### Study Population and Clinical Procedures

**Figure 1.**
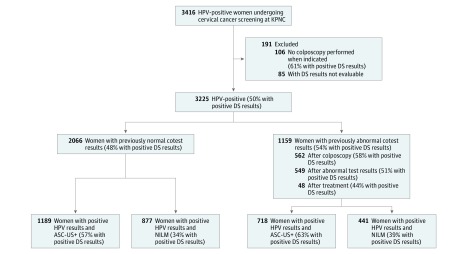
CONSORT Diagram ASC-US+ indicates atypical squamous cells of undetermined significance or worse; DS, dual stain; HPV, human papillomavirus; KPNC, Kaiser Permanente Northern California; and NILM, negative for intraepithelial lesions or malignant neoplasm.

The study was conducted within the routine cervical cancer screening program at KPNC. Per KPNC guidelines, women with negative HPV and Papanicolaou cytologic (cotest) results should return for regular screening after 3 years. Women with HPV-positive atypical squamous cells of undetermined significance (ASC-US), low-grade squamous intraepithelial lesions, or more severe cytologic results are referred to undergo immediate colposcopy. Women with positive HPV results and negative cytologic results should undergo repeat cotesting after 1 year and are referred to undergo colposcopy when either HPV or Papanicolaou cytologic results (at a threshold of ASC-US or worse [ASC-US+]) are positive. Women with negative results of a repeat cotest are not referred to undergo colposcopy. Per KPNC recommendations, all women undergoing colposcopy should receive at least 1 biopsy. Pathologists evaluating cervical biopsies routinely had access to patients’ full medical records. Clinical management was not based on results of DS or HPV16/18 testing. In total, 3416 HPV-positive women who underwent cotesting at KPNC between September 16 and October 31, 2015, were included in the study. Follow-up for cervical precancer and cancer end points was conducted using electronic medical records through December 31, 2018. We excluded 85 women without evaluable DS results and 106 women without colposcopy when indicated per the algorithms described above (Figure 1). We evaluated the performance of DS and partial genotyping in the following 2 populations: (1) all HPV-positive women who underwent cotesting for screening, triage, surveillance, and management, representing all women currently undergoing cotesting at KPNC (n = 3225); and (2) the subset of HPV-positive women without abnormal cotest results in the preceding 42 months, representing a screening population of women who are identified to be newly HPV-positive (n = 2066). The study was approved by the KPNC Institutional Review Board and was exempted from institutional review at the National Cancer Institute by the Office of Human Subjects Research. Patient consent was waived because deidentified discarded specimens were used in this study.

### Clinical Routine HPV Testing and Cytologic Screening

Hybrid capture 2 (HC2 High-Risk HPV DNA Test; Qiagen Inc) was conducted on specimen transport medium specimens per the manufacturer’s instructions. Papanicolaou cytologic samples were collected in SurePath (Becton Dickinson) fixative. SurePath slides were prepared, stained, and processed on the BD FocalPoint Slide Profiler (Becton Dickinson). Medical history, HPV test results, and FocalPoint results were transmitted to the cytotechnologists reviewing slides on guided screening microscopes. All slides from HPV-positive women were evaluated by guided screening–assisted screening and full manual review. All abnormal slides were sent for pathology review. In addition, all negative Papanicolaou cytologic slides from HPV-positive women were rescreened manually.

### DS and cobas 4800 Testing

SurePath tubes containing the residual pellet after preparation of routine Papanicolaou cytologic slides from HPV-positive women with final Papanicolaou cytologic results were removed consecutively from storage. SurePath slides were prepared by an experienced laboratory assistant. The prepared slides underwent staining by a histology technician who successfully completed CINtec PLUS (Roche Diagnositics) DS training, using the CINtec PLUS kit following the manufacturer’s instructions. The DS cytologic slides were evaluated by cytologists after a 2-day training and certification to evaluate p16/Ki-67 DS cytologic slides. The cytotechnologists marked at least 1 positive cell on any DS-positive slides and documented their results electronically. The pathologists reviewed all slides and documented their results electronically. Slides with at least 1 DS-positive cell were considered positive.

The residual matching specimen transport medium tubes were removed from frozen storage and processed on the cobas 4800 instrument (Roche) by licensed and trained personnel per the manufacturer’s protocol. Results from cobas testing are either HPV16/18 positive, other 12 high-risk HPV positive, or HPV negative.

### Statistical Analysis

We evaluated the distribution of HPV16/18 and other high-risk HPV types (high-risk HPV12), and positive DS results by baseline cytologic findings and poorest histologic test results. Cytologic findings were classified by the Bethesda System^16^: negative for intraepithelial lesions or malignant neoplasm (NILM); ASC-US; low-grade squamous intraepithelial lesions; atypical squamous cells, cannot rule out high-grade squamous intraepithelial lesion; and high-grade squamous intraepithelial lesions. Final diagnosis was established by histopathologic findings classified according to the cervical intraepithelial neoplasia (CIN) scale: no indication for biopsy, normal, other (including atypia), CIN grade 1 (CIN1), CIN grade 2 (CIN2), CIN grade 3 (CIN3) and adenocarcinoma in situ, or cancer. Women who did not have an indication for colposcopy (“no indication for biopsy,” eg, because of a negative results on a repeat cotest) were considered free of disease. The primary end point included CIN3, adenocarcinoma in situ, and cancer (CIN3+). The secondary end point included CIN2, CIN3, adenocarcinoma in situ, and cancer (CIN2+). We calculated sensitivity, specificity, positive predictive value, and negative predictive value for detection of CIN2+ and CIN3+ for primary HPV screening with partial genotyping and DS triage, and for DS triage in HPV-positive women without partial genotyping. The strategy currently approved by the US Food and Drug Administration (FDA) of primary HPV screening with partial genotyping and cytologic triage was the reference for the comparisons.

Differences in positivity, sensitivity, and specificity were evaluated using an exact McNemar χ^2^ and differences in predictive values were evaluated using the method developed by Leisenring et al^17^ using the R package, DTComPair (R Foundation for Statistical Computing). In a supplemental analysis, we stratified clinical performance measures by age groups (<29, 30-34, 35-44, and ≥45 years). We evaluated absolute risks of CIN2+ and CIN3+ in strata by cytologic testing, HPV16/18 genotyping, and DS. Exact binomial 95% CIs were calculated for proportions. We calculated the absolute risk of CIN3+for the currently approved primary HPV screening strategy with partial genotyping and cytologic triage, for DS in combination with partial genotyping, and for DS alone without partial genotyping (eFigure 1 in the Supplement). For each test result, we plotted the absolute risk of CIN3+ in relation to internal risk benchmarks derived from this study population: the risk of CIN3+ during the observation period in women with positive HPV test results and ASC-US (5.7% [53 of 927]) was the benchmark for immediate referral to undergo colposcopy and the risk of CIN3+ for women with positive HPV test results and NILM (2.8% [37 of 1318]) was the benchmark for a repeat cotest in 1 year. To evaluate the clinical efficiency of each strategy shown in eFigure 1 in the Supplement, we calculated clinical estimates (assuming full compliance with recommendations by patients and providers) of the number of women referred to undergo colposcopy immediately, the number of women recommended for 1-year repeat testing (according to the FDA-approved primary HPV testing algorithm), the number of women referred to undergo colposcopy after 1-year repeat screening (based on estimates from women with positive HPV test results and NILM, 549 of 1097 [50.0%] have positive HPV test results after 1 year), the number of CIN3+ cases detected, and the ratio of the number of tests and colposcopies per case of CIN3+ detected. Analyses were performed in R, version 3.3.1. All *P* values were from 2-sided tests and results were deemed statistically significant at *P* < .05.

## Results

### DS Positivity by Cytologic and Histologic Results

Of 3225 women included in this study, 3 had cancer, 233 had CIN3 or adenocarcinoma in situ, 236 had CIN2, 2056 had histologic findings less severe than CIN2, and 697 did not have an indication for colposcopy (Table 1). Positive DS results increased from 36.1% (476 of 1318) in women with NILM cytologic findings to 96.0% (96 of 100) in women with high-grade squamous intraepithelial lesions, and from 32.9% (229 of 697) in women without biopsy to 88.4% (206 of 233) in women with CIN3 or adenocarcinoma in situ.

**Table 1.** HPV16/18 and DS Positivity by Histologic Test Results and Cytologic Test Results Among 3225 HPV-Positive Women^a^

Cytologic Test Result	Women, No. (%)							
	Total (N = 3225)	No Biopsy (n = 697)	Benign (n = 652)	Other (n = 79)	CIN1 (n = 1325)	CIN2 (n = 236)	CIN3 or AIS (n = 233)	Cancer (n = 3)
NILM	1318 (40.9)	636 (91.2)	273 (41.9)	24 (30.4)	296 (22.3)	52 (22.0)	37 (15.9)	0
Positive HPV16/18 result	221 (16.8)	100 (15.7)	43 (15.8)	3 (12.5)	37 (12.5)	18 (34.6)	20 (54.1)	0
Positive HR HPV12 result	871 (66.1)	426 (67.0)	175 (64.1)	18 (75.0)	213 (72.0)	24 (46.2)	15 (40.5)	0
Positive DS result	476 (36.1)	202 (31.8)	88 (32.2)	9 (37.5)	123 (41.6)	29 (55.8)	25 (67.6)	0
ASC-US	927 (28.7)	34 (4.9)	237 (36.3)	33 (41.8)	504 (38.0)	66 (28.0)	53 (22.7)	0
Positive HPV16/18 result	173 (18.7)	3 (8.8)	37 (15.6)	5 (15.2)	80 (15.9)	22 (33.3)	26 (49.1)	0
Positive HR HPV12 result	600 (64.7)	23 (67.6)	153 (64.6)	25 (75.8)	340 (67.5)	38 (57.6)	21 (39.6)	0
Positive DS result	462 (49.8)	14 (41.2)	99 (41.8)	12 (36.4)	244 (48.4)	50 (75.8)	43 (81.1)	0
LSIL	723 (22.4)	24 (3.4)	119 (18.2)	21 (26.6)	452 (34.1)	65 (27.5)	42 (18.0)	0
Positive HPV16/18 result	120 (16.6)	3 (12.5)	12 (10.1)	2 (9.5)	65 (14.4)	16 (24.6)	22 (52.4)	0
Positive HR HPV12+ result	486 (67.2)	17 (70.8)	84 (70.6)	16 (76.2)	307 (67.9)	43 (66.2)	19 (45.2)	0
Positive DS+ result	437 (60.4)	11 (45.8)	62 (52.1)	15 (71.4)	258 (57.1)	53 (81.5)	38 (90.5)	0
ASC-H	157 (54.9)	3 (0.4)	15 (2.3)	0	59 (4.5)	36 (15.2)	44 (18.9)	0
Positive HPV16/18 result	57 (36.3)	1 (33.3)	4 (26.7)	0	16 (27.1)	16 (44.4)	20 (45.5)	0
Positive HR HPV12 result	80 (51.0)	1 (33.3)	7 (46.7)	0	38 (64.4)	15 (41.7)	19 (43.2)	0
Positive DS+ result	139 (88.5)	2 (66.7)	12 (80.0)	0	48 (81.4)	33 (91.7)	44 (100)	0
HSIL	100 (3.1)	0	8 (1.2)	1 (1.3)	14 (1.1)	17 (7.2)	57 (24.5)	3 (100)
Positive HPV16/18 result	52 (52.0)	0	2 (25.0)	1 (100)	5 (35.7)	11 (64.7)	31 (54.4)	2 (66.7)
Positive HR HPV12 result	36 (36.0)	0	3 (37.5)	0	7 (50.0)	6 (35.3)	19 (33.3)	1 (33.3)
Positive DS result	96 (96.0)	0	7 (87.5)	1 (100)	12 (85.7)	17 (100)	56 (98.2)	3 (100)
Total	3225 (100)	697 (21.6)	652 (20.2)	79 (2.4)	1325 (41.1)	236 (7.3)	233 (7.2)	3 (0.09)
Positive HPV16/18 result	623 (19.3)	107 (15.4)	98 (15.0)	11 (13.9)	203 (15.3)	83 (35.2)	119 (51.1)	2 (66.7)
Positive HR HPV12 result	2073 (64.3)	467 (67.0)	422 (64.7)	59 (74.7)	905 (68.3)	126 (53.4)	93 (39.9)	1 (33.3)
Positive DS result	1610 (49.9)	229 (32.9)	268 (41.1)	37 (46.8)	685 (51.7)	182 (77.1)	206 (88.4)	3 (100)

Abbreviations: AIS, adenocarcinoma in situ; ASC-H, atypical squamous cells, cannot rule out high-grade squamous intraepithelial lesion; ASC-US, atypical squamous cells of undetermined significance; CIN, cervical intraepithelial neoplasia (grades 1-3); DS, dual stain cytologic test; HSIL, high-grade squamous intraepithelial lesions; HPV, human papillomavirus; HPV16/18+, positive for either HPV16 or HPV18; HR HPV12+, positive for at least 1 of the other high-risk HPV types; LSIL, low-grade squamous intraepithelial lesions; NILM, negative for intraepithelial lesion or malignant neoplasm; No Biopsy, No indication for colposcopy/biopsy.

^a^ The percentage in the first row in each cytology category gives the total across all cytology groups; the other percentages are within the cytology category.

### Cytologic and DS Triage for Detecting Precancerous Lesions

Compared with the currently approved primary HPV screening strategy with partial genotyping and cytologic triage, a combination of DS with HPV16/18 genotyping had significantly lower positivity than cytologic testing alone (1818 [56.4%; 95% CI, 54.6%-58.1%] vs 2128 [66.0%; 95% CI, 64.3%-67.6%]; *P* < .001), while the sensitivity was equal for both strategies (Table 2). Although DS alone had a slightly reduced sensitivity compared with the combined strategies, the sensitivity was higher than that with cytologic testing alone and the specificity was significantly higher compared with all other strategies. These results were consistent across all age groups (eTable 1 in the Supplement). Similar patterns were observed for detection of CIN2+ (Table 2). For triage of HPV-positive women with partial genotyping, DS showed better risk stratification for CIN3+ than did Papanicolaou cytologic testing, with women with positive DS results having a higher risk than women with positive Papanicolaou test results for CIN3+ (218 of 1818 [12.0%; 95% CI, 10.5%-13.5%] vs 219 of 2128 [10.3%; 95% CI, 9.0%-11.6%]; *P* = .005) (Table 3).

**Table 2.** Performance of Cytologic Testing, Dual Stain, HPV16/18 Testing, and Combinations Among 3225 HPV-Positive Women to Detect CIN3+ and CIN2+

Characteristic	Women, No./Total No. (%) [95% CI]			
	HPV16/18 and Cytologic Findings	HPV16/18 and Dual Stain Results	Cytologic Findings	Dual Stain Results
Threshold	Either ASC-US+ or positive results for HPV16 or HPV18	1 Cell positive on dual stain or positive results for HPV16 or HPV18	ASC-US+	1 Cell positive on dual stain
Positivity	2128/3225 (66.0) [64.3-67.6]	1818/3225 (56.4) [54.6-58.1]^a^	1907/3225 (59.1) [57.4-60.8]^a^	1610/3225 (49.9) [48.2-51.7]^a^
Detection of CIN3+ (n = 236)				
Sensitivity	219/236 (92.8) [89.5-96.1]	218/236 (92.4) [89.0-95.8]	199/236 (84.3) [79.7-89.0]^a^	209/236 (88.6) [84.5-92.6]^b^
Specificity	1080/2989 (36.1) [34.4-37.9]	1389/2989 (46.5) [44.7-48.3]^a^	1281/2989 (42.9) [41.1-44.6]^a^	1588/2989 (53.1) [51.3-54.9]^a^
PPV	219/2128 (10.3) [9.0-11.6]	218/1818 (12.0) [10.5-13.5]^a^	199/1907 (10.4) [9.1-11.8]	209/1610 (13.0) [11.3-14.6]^a^
NPV	1080/1097 (98.5) [97.7-99.2]	1389/1407 (98.7) [98.1-99.3]	1281/1318 (97.2) [96.3-98.1]^a^	1588/1615 (98.3) [97.7-99.0]
Detection of CIN2+ (n = 472)				
Sensitivity	421/472 (89.2) [86.4-92.0]	417/472 (88.3) [85.5-91.2]	383/472 (81.1) [77.6-84.7]^a^	391/472 (82.8) [79.4-86.2]^a^
Specificity	1046/2753 (38.0) [36.2-39.8]	1352/2753 (49.1) [47.2-51.0]^a^	1229/2753 (44.6) [42.8-46.5]^a^	1534/2753 (55.7) [53.9-57.6]^a^
PPV	421/2128 (19.8) [18.1-21.4]	417/1818 (22.9) [21.0-24.9]^a^	383/1907 (20.1) [18.3-21.9]	391/1610 (24.3) [22.2-26.4]^a^
NPV	1046/1097 (95.4) [94.1-96.6]	1352/1407 (96.1) [95.1-97.1]	1229/1318 (93.2) [91.9-94.6]^a^	1534/1615 (95.0) [93.9-96.0]

Abbreviations: ASC-US+, Atypical Squamous Cells of Undetermined Significance or more severe cytologic diagnosis; CIN2+, cervical intraepithelial neoplasia grade 2 or worse; CIN3+, cervical intraepithelial neoplasia grade 3 or worse; HPV, human papillomavirus; NPV, negative predictive value; PPV, positive predictive value.

^a^ *P* < .001 compared with cytologic findings and positive HPV16/18 results calculated using McNemar test for positivity and sensitivity and specificity and the Leisenring method for predictive values.

**Table 3.** Colposcopy Referral and CIN3+ Detection for 3 Different Combined Screening and Triage Strategies

Screening and Triage Approach	Immediate Colposcopy	1-y Return	Immediate Colposcopy		No. of Colposcopies per CIN3+ Detected	No. of Women		No. of CIN3+ Detected After 1-y Colposcopy	No. of Colposcopies	
			No. of Women^a^	No. of CIN3+ Detected		1-y Rescreening	Colposcopy After Rescreening		Per CIN3+ Detected After 1 y	Total
FDA-approved HPV screening and partial genotyping with cytologic triage	All women with positive HPV16/18 results; women with positive HPV results and negative HPV16/18 results with ASC-US+	Women with negative HPV16/18 results with NILM	2128	219	9.7	1097	549	17	32.3	2677
Primary HPV testing with partial genotyping and dual stain triage	Option 1: all women with positive HPV16/18-results; all women with positive dual stain results	NA	1818	218	8.3	0	NA	NA	NA	1818
	Option 2: all women with positive dual stain results	Women with positive HPV16/18 results, women with negative dual stain results	1610	209	7.7	208	128	9	14.2	1738
Primary HPV testing with dual stain triage	All women with positive dual stain results	NA	1610	209	7.7	0	NA	NA	NA	1610

Abbreviations: ASC-US+, atypical squamous cells of undetermined significance or worse; CIN3+, cervical intraepithelial neoplasia grade 3 or worse; FDA, US Food and Drug Administration; HV, human papillomavirus; HSIL, high-grade squamous intraepithelial lesions; LSIL, low-grade squamous intraepithelial lesions; NA, not applicable; NILM, negative for intraepithelial lesion or malignant neoplasm.

^a^ Women with HPV-negative LSIL or HPV-negative HSIL are not included. The number of women who underwent colposcopy after rescreening is estimated to be about 50% of women with an HPV-positive result at their repeat cotest.

### Cervical Precancerous Lesion Risk in Combinations of DS, Positive HPV16/18 Results, and Cytologic Testing

**Figure 2.**
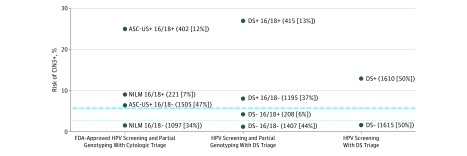
Risk of Cervical Intraepithelial Neoplasia Grade 3 or Worse (CIN3+) in Strata of Cytologic Testing, Dual Stain (DS), and Human Papillomavirus (HPV)16/18 The risk of CIN3+ for combinations of cytologic testing with HPV16/18 genotyping, DS with HPV16/18 genotyping, and DS alone is plotted on the y-axis, with number and percentage of women for specific test combinations indicated. The dotted line corresponds to the 1-year return threshold (HPV-positive negative for intraepithelial lesions or malignant neoplasm [NILM], 2.8%), and the dashed line corresponds to the colposcopy referral threshold (HPV-positive atypical squamous cells of undetermined significance [ASC-US], 5.7%). FDA indicates US Food and Drug Administration.

Figure 2 shows the risk of CIN3+ among HPV-positive women for the currently approved primary HPV screening strategy with partial genotyping and cytologic triage, for DS in combination with partial genotyping, and for DS alone without partial genotyping in relation to clinical management thresholds. The strategy of partial genotyping with DS yielded better risk stratification compared with the current standard: more women had a very low risk (1407 [43.6%] vs 1097 [34.0%]), and fewer women would be referred to undergo colposcopy immediately (1610 [49.9%] vs 2128 [66.0%]). More important, while women with NILM cytologic findings and positive HPV16/18 results had a risk high enough for colposcopy referral, the risk in women with negative DS results and positive HPV16/18 results was clearly below the threshold for colposcopy referral. Women with negative DS results and negative HPV16/18 results had the lowest risk of all combinations. The strategy of DS alone without partial genotyping provided good risk stratification, with half the women under the threshold for return testing at 1 year and the other half clearly above the threshold for referral to undergo colposcopy. The addition of partial genotyping to DS did not increase the percentage of women above the threshold for referral to undergo colposcopy. The individual risk estimates for all combinations of cytologic testing, HPV16/18 genotyping, and DS results are summarized in eTable 2 in the Supplement.

### Detection in Women Without Previous Abnormal Cotest Results

Among the 2066 women without previous abnormal cotest results, the results with respect to positive DS and HPV16/18 positivity (eTable 3 in the Supplement), and the performance of these assays in comparison with the approved primary HPV screening strategy (eTable 4 in the Supplement) were similar to the overall population. Risks in strata of cytologic testing, HPV genotyping, and DS were largely similar to risks observed in the overall population (eTable 5 in the Supplement). Compared with the overall population of HPV-positive women, we observed very similar risk stratification of the approved strategy and the DS strategies in relation to clinical management thresholds, with one notable difference being the lower risk observed in women with negative DS results and positive HPV16/18 results, which was below the 1-year return threshold (eFigure 2 in the Supplement).

### Efficiency of Primary HPV Screening With DS Compared With Cytologic Triage

The currently approved HPV screening strategy had the highest immediate referrals to undergo colposcopy (2128 [66.0%]) and overall referrals to undergo colposcopy (2677 [83.0%]). This strategy required 9.7 colposcopies to detect CIN3+ findings at immediate referral. All DS strategies required substantially fewer colposcopies, from 1818 for the combined partial genotyping and DS strategy in which all women with positive results for either of the tests are referred to undergo colposcopy (a 32.1% [859 of 2677] reduction), to 1610 for DS alone, requiring between 8.3 and 7.7 colposcopies per case of CIN3+ detected. The currently approved HPV screening strategy requires retesting of women with positive HPV results and negative HPV16/18 NILM cytologic results after 1 year. Of these women, half have positive HPV or cytologic results at the 1-year visit and require colposcopy referral per current recommendations. Among women undergoing primary HPV screening with cytologic triage who are referred to undergo colposcopy after a repeat cotest, only 17 additional cases of CIN3 were detected in 549 colposcopies, requiring 32.2 colposcopies for each case of CIN3+ detected. In contrast, 1-year retesting of women with positive HPV16/18 results and negative DS results required 14.2 colposcopies per case of CIN3+ detected.

## Discussion

Cervical cancer screening is transitioning from cytologic testing to primary HPV testing. Although a negative HPV test result provides reassurance against prevalent precancerous lesions or cancer, most HPV-positive women have transient infections that are not associated with cervical precancerous lesions, highlighting the need for additional triage tests. The currently approved algorithm for primary HPV screening in the United States includes partial genotyping and cytologic testing for triage of HPV-positive women.^6,7^ Within KPNC, this strategy would lead to two-thirds of HPV-positive women being referred to undergo colposcopy immediately, and more than 80% total of HPV-positive women being referred to undergo colposcopy immediately after 1 year. The strategy creates a substantial burden for women and has implications for health care infrastructure and cost, because most referrals to under colposcopy do not lead to detection of precancerous lesions.

A previous study reported that DS has higher sensitivity and specificity compared with cytologic testing for triage of HPV-positive women.^13^ In the current study, we evaluated the programmatic clinical performance of DS in conjunction with partial genotyping, allowing a direct comparison of a novel DS and HPV16/18 algorithm with the currently approved approach for triage of HPV-positive women. Using a risk-centered approach with internal benchmarks based on current management,^18^ we found that performing DS would accurately identify most women at very low risk of precancerous lesions (negative HPV16/18 results and negative DS results) who may safely undergo retesting at extended intervals, a small group of women with somewhat elevated risk that is not high enough for colposcopy referral (positive HPV16/18 results and negative DS results), and the remaining women whose risk is well above the threshold for referral to undergo colposcopy (all women with positive DS results with highest risk among the women with positive DS results and positive HPV16/18 results). Women with normal cytologic test results, but positive HPV16/18 results, have a risk above the threshold for referral to undergo colposcopy, much higher than the risk of women with negative DS results and positive HPV16/18 results. Dual stain alone, without genotyping, provides very similar risk stratification, indicating that DS is also effective for triage of HPV screening tests that do not provide genotyping. Furthermore, the risk stratification of DS among HPV16/18–negative women is better compared with Papanicolaou cytologic testing, suggesting that DS is a good triage option for vaccinated populations that have reduced prevalence of HPV16/18 infections.

We evaluated the approved primary HPV screening strategy and novel DS-based triage strategies with respect to colposcopy referral and disease detection. We found that DS-based strategies were more efficient (indicated by lower numbers of colposcopies needed per case of CIN3+ detected) compared with cytologic testing–based strategies. Our data show the poor efficiency of 1-year repeat testing in currently approved strategies, with large numbers of colposcopies after repeat testing yielding only very few cases of CIN3+. Compared with a combined DS and HPV16/18 genotyping approach, the few additional cases of CIN3+ that are detected by these strategies involving 1-year repeat testing have negative HPV16/18 results and negative DS results at baseline, suggesting that they are small, low-risk precancerous lesions that will either resolve or be safely detected at the next 3-year screening visit.

We also found that primary HPV with DS cytologic testing can supplant a high-quality cotesting program and provide sensitive detection of cervical precancerous lesions while referring fewer women to undergo colposcopy. Dual stain cytologic testing showed improved performance compared with Papanicolaou cytologic testing in both the subset of women undergoing routine screening and the full population including women undergoing cotests for management of abnormal screening results, after colposcopy, or after treatment. Long-term follow-up from a previous study shows that women with negative DS results have a low risk of cervical precancerous lesions over 5 years.^12^ These data, combined with the low risk estimates in the current study for women with negative DS results, support the finding that retesting intervals in women with negative DS results can be safely extended to 3 years.

### Strengths and Limitations

Our study has several strengths. We evaluated a large population with uniform and well-organized screening and management procedures, good disease ascertainment, and limited loss to follow-up. Our large population provides precise risk estimates for various combinations of test results. Furthermore, the risk levels from KPNC for different combinations of cytologic testing and HPV testing were the basis of current US screening and management guidelines,^2,19^ allowing us to directly compare DS results with established management thresholds. All study procedures were implemented at the KPNC regional laboratory and performed in parallel with routine clinical operations, showing the feasibility of an integrated HPV screening and DS triage program in a clinical laboratory. A previous study showed the high reproducibility of DS when read by experienced cytotechnologists.^20^ Currently, approaches for automated evaluation of DS slides are being developed based on digital imaging; these approaches may further improve reproducibility, accuracy, and workflow of DS cytologic testing.^21^

Limitations of our study should be noted. Papanicolaou cytologic testing at KPNC may be more sensitive than Papanicolaou cytologic testing performed at other health facilities. Thus, the specific performance comparisons between DS and Papanicolaou may differ in other settings. In our observational study, clinical management was based on HPV cytologic cotesting, with differential follow-up of women with positive HPV test results and positive cytologic results and women with positive HPV test results and negative cytologic results (repeat testing after 1 year). However, we had high completion rates of follow-up procedures. Furthermore, management guidelines were not always followed exactly, reflecting real-life practice in routine cervical cancer screening programs rather than in a clinical trial. Follow-up is ongoing; it will be important to determine how long a negative DS test result in combination with HPV16/18 genotyping is associated with a low risk of cervical precancerous lesions.

## Conclusions

We found that, for primary HPV screening, DS has both higher sensitivity and specificity compared with cytologic testing for triage of HPV-positive women. Because of the greater reassurance of negative DS results, screening intervals can be extended compared with the screening intervals after negative cytologic results. Dual stain reduces unnecessary colposcopy referral and unnecessary cervical biopsies, and may reduce unnecessary treatment compared with Papanicolaou cytologic testing. Our estimates of sensitivity, absolute risk, and colposcopy referral for various triage strategies can guide implementation of primary HPV screening.

## Notes

Supplement. **eTable 1.** Clinical Performance of Cytology, Dual Stain, and HPV16/18 Testing in Age Groups **eTable 2.** Risk of CIN3+ and CIN2+ in Strata of Cytology, HPV16/18 Genotype, and Dual Stain Results **eTable 3.** HPV16/18 and p16/Ki-67 Positivity by Histology and Cytology Results in 2066 Women Without Known Preceding Abnormal Co-Test **eTable 4.** Performance of Cytology, Dual Stain, HPV16/18 and Combinations Among 2066 HPV-Positive Women Without Preceding Abnormal Co-Test to Detect CIN3+ (n = 131) and CIN2+ (n = 277) **eTable 5.** Risk of CIN3+ and CIN2+ in Strata of Cytology, HPV16/18 Genotype and Dual Stain Results in Women With Preceding Normal Co-Tests **eFigure 1.** Screening and Triage Algorithms **eFigure 2**. Risk of CIN3+ in Strata of Cytology, Dual Stain, and HPV16/18 Among Women With Normal Preceding Co-Test

## References

[1] Wentzensen N, Arbyn M, Berkhof J, . Eurogin 2016 roadmap: how HPV knowledge is changing screening practice. Int J Cancer. 2017;140(10):2192-2200. doi:10.1002/ijc.30579 28006858

[2] Gage JC, Schiffman M, Katki HA, . Reassurance against future risk of precancer and cancer conferred by a negative human papillomavirus test. J Natl Cancer Inst. 2014;106(8):dju153. doi:10.1093/jnci/dju153 25038467 PMC4111283

[3] Wentzensen N. Triage of HPV-positive women in cervical cancer screening. Lancet Oncol. 2013;14(2):107-109. doi:10.1016/S1470-2045(12)70568-5 23261357 PMC4198376

[4] Cuschieri K, Ronco G, Lorincz A, . Eurogin roadmap 2017: triage strategies for the management of HPV-positive women in cervical screening programs. Int J Cancer. 2018;143(4):735-745. doi:10.1002/ijc.31261 29341110

[5] Wentzensen N, Schiffman M, Palmer T, Arbyn M. Triage of HPV positive women in cervical cancer screening. J Clin Virol. 2016;76(suppl 1):S49-S55. doi:10.1016/j.jcv.2015.11.015 26643050 PMC4789103

[6] Huh WK, Ault KA, Chelmow D, . Use of primary high-risk human papillomavirus testing for cervical cancer screening: interim clinical guidance. J Low Genit Tract Dis. 2015;19(2):91-96. doi:10.1097/LGT.0000000000000103 25574659

[7] Wright TC, Stoler MH, Behrens CM, Sharma A, Zhang G, Wright TL. Primary cervical cancer screening with human papillomavirus: end of study results from the ATHENA study using HPV as the first-line screening test. Gynecol Oncol. 2015;136(2):189-197. doi:10.1016/j.ygyno.2014.11.076 25579108

[8] Guan P, Howell-Jones R, Li N, . Human papillomavirus types in 115,789 HPV-positive women: a meta-analysis from cervical infection to cancer. Int J Cancer. 2012;131(10):2349-2359. doi:10.1002/ijc.27485 22323075

[9] Wright TC Jr, Stoler MH, Behrens CM, Sharma A, Sharma K, Apple R. Interlaboratory variation in the performance of liquid-based cytology: insights from the ATHENA trial. Int J Cancer. 2014;134(8):1835-1843. doi:10.1002/ijc.28514 24122508

[10] Carozzi F, Confortini M, Dalla Palma P, ; New Technologies for Cervival Cancer Screening (NTCC) Working Group. Use of p16-INK4A overexpression to increase the specificity of human papillomavirus testing: a nested substudy of the NTCC randomised controlled trial. Lancet Oncol. 2008;9(10):937-945. doi:10.1016/S1470-2045(08)70208-0 18783988

[11] Carozzi F, Gillio-Tos A, Confortini M, ; NTCC working group. Risk of high-grade cervical intraepithelial neoplasia during follow-up in HPV-positive women according to baseline p16-INK4A results: a prospective analysis of a nested substudy of the NTCC randomised controlled trial. Lancet Oncol. 2013;14(2):168-176. doi:10.1016/S1470-2045(12)70529-6 23261355

[12] Clarke MA, Cheung LC, Castle PE, . Five-year risk of cervical precancer following p16/Ki-67 dual-stain triage of HPV-positive women [published online October 11, 2018]. JAMA Oncol. doi:10.1001/jamaoncol.2018.4270 30325982 PMC6439556

[13] Wentzensen N, Fetterman B, Castle PE, . p16/Ki-67 Dual stain cytology for detection of cervical precancer in HPV-positive women. J Natl Cancer Inst. 2015;107(12):djv257. doi:10.1093/jnci/djv257 26376685 PMC4675094

[14] Wentzensen N, Schwartz L, Zuna RE, . Performance of p16/Ki-67 immunostaining to detect cervical cancer precursors in a colposcopy referral population. Clin Cancer Res. 2012;18(15):4154-4162. doi:10.1158/1078-0432.CCR-12-0270 22675168 PMC4237612

[15] Wright TC Jr, Behrens CM, Ranger-Moore J, . Triaging HPV-positive women with p16/Ki-67 dual-stained cytology: results from a sub-study nested into the ATHENA trial. Gynecol Oncol. 2017;144(1):51-56. doi:10.1016/j.ygyno.2016.10.031 28094038

[16] Solomon D, Davey D, Kurman R, ; Forum Group Members; Bethesda 2001 Workshop. The 2001 Bethesda System: terminology for reporting results of cervical cytology. JAMA. 2002;287(16):2114-2119. doi:10.1001/jama.287.16.2114 11966386

[17] Leisenring W, Alonzo T, Pepe MS. Comparisons of predictive values of binary medical diagnostic tests for paired designs. Biometrics. 2000;56(2):345-351. doi:10.1111/j.0006-341X.2000.00345.x 10877288

[18] Katki HA, Schiffman M, Castle PE, . Benchmarking CIN 3+ risk as the basis for incorporating HPV and Pap cotesting into cervical screening and management guidelines. J Low Genit Tract Dis. 2013;17(5)(suppl 1):S28-S35. doi:10.1097/LGT.0b013e318285423c 23519302 PMC3616419

[19] Katki HA, Kinney WK, Fetterman B, . Cervical cancer risk for women undergoing concurrent testing for human papillomavirus and cervical cytology: a population-based study in routine clinical practice. Lancet Oncol. 2011;12(7):663-672. doi:10.1016/S1470-2045(11)70145-0 21684207 PMC3272857

[20] Wentzensen N, Fetterman B, Tokugawa D, . Interobserver reproducibility and accuracy of p16/Ki-67 dual-stain cytology in cervical cancer screening. Cancer Cytopathol. 2014;122(12):914-920. doi:10.1002/cncy.21473 25132656

[21] Grabe N, Lahrmann B, Pommerencke T, von Knebel Doeberitz M, Reuschenbach M, Wentzensen N. A virtual microscopy system to scan, evaluate and archive biomarker enhanced cervical cytology slides. Cell Oncol. 2010;32(1-2):109-119. doi:10.3233/CLO-2009-0508 20208139 PMC4237613

