# Supplementary material for: Clinical Evaluation of Human Papillomavirus Screening With p16/Ki-67 Dual Stain Triage in a Large Organized Cervical Cancer Screening Program
Source: JAMA Intern Med. 2019 May 13;179(7):1–9. doi: 10.1001/jamainternmed.2019.0306 (PMC6515572; doi:10.1001/jamainternmed.2019.0306)
Supplement: Supplement. — eTable 1. Clinical Performance of Cytology, Dual Stain, and HPV16/18 Testing in Age Groups eTable 2. Risk of CIN3+ and CIN2+ in Strata of Cytology, HPV16/18 Genotype, and Dual Stain Results eTable 3. HPV16/18 and p16/Ki-67 Positivity by Histology and Cytology Results in 2066 Women Without Known Preceding Abnormal Co-Test eTable 4. Performance of Cytology, Dual Stain, HPV16/18 and Combinations Among 2066 HPV-Positive Women Without Preceding Abnormal Co-Test to Detect CIN3+ (n = 131) and CIN2+ (n = 277) eTable 5. Risk of CIN3+ and CIN2+ in Strata of Cytology, HPV16/18 Genotype and Dual Stain Results in Women With Preceding Normal Co-Tests eFigure 1. Screening and Triage Algorithms eFigure 2. Risk of CIN3+ in Strata of Cytology, Dual Stain, and HPV16/18 Among Women With Normal Preceding Co-Test [file jamainternmed-e190306-s001.pdf]

Supplementary Online Content

Wentzensen N, Clarke MA, Bremer R, et al. Clinical evaluation of human papillomavirus screening with p16/Ki-67 dual stain triage in a large organized cervical cancer screening program. *JAMA Intern Med.* Published online May 13, 2019.
doi:10.1001/jamainternmed.2019.0306

eTable 1. Clinical Performance of Cytology, Dual Stain, and HPV16/18 Testing in Age Groups
eTable 2. Risk of CIN3+ and CIN2+ in Strata of Cytology, HPV16/18 Genotype, and Dual Stain Results
eTable 3. HPV16/18 and p16/Ki-67 Positivity by Histology and Cytology Results in 2066 Women Without Known Preceding Abnormal Co-Test
eTable 4. Performance of Cytology, Dual Stain, HPV16/18 and Combinations Among 2066 HPV-Positive Women Without Preceding Abnormal Co-Test to Detect CIN3+ (n=131) and CIN2+ (n=277)
eTable 5. Risk of CIN3+ and CIN2+ in Strata of Cytology, HPV16/18 Genotype and Dual Stain Results in Women With Preceding Normal Co-Tests
eFigure 1. Screening and Triage Algorithms
eFigure 2. Risk of CIN3+ in Strata of Cytology, Dual Stain, and HPV16/18 Among Women With Normal Preceding Co-Test

This supplementary material has been provided by the authors to give readers additional information about their work.

© 2019 Wentzensen N et al. *JAMA Internal Medicine*.

eTable 1. Clinical Performance of Cytology, Dual Stain, and HPV16/18 Testing in Age Groups

	**Cytology (95% CI)**	**Dual stain (95% CI)**	**HPV16/18 (95% CI)**
**Threshold**	ASC-US+	1 dual stain positive cell	Either HPV16 or HPV18 positive
**<29 years (998 women, 80 CIN3+)**			
**Positivity**	63.9%	52.6%	19.7%
**Sensitivity**	86.3%	86.3%	55.0%
**Specificity**	38.0%	50.4%	83.6%
**PPV**	10.8%	13.2%	22.6%
**NPV**	96.9%	97.7%	95.5%
**30-34 years (673 women, 66 CIN3+)**			
**Positivity**	58.8%	51.2%	21.1%
**Sensitivity**	86.4%	87.9%	54.6%
**Specificity**	44.2%	52.9%	82.5%
**PPV**	14.4%	16.9%	25.4%
**NPV**	96.8%	97.6%	94.4%
**35-44 years (719 women, 48 CIN3+)**			
**Positivity**	55.4%	44.7%	20.1%
**Sensitivity**	79.2%	85.4%	41.7%
**Specificity**	46.4%	58.1%	81.7%
**PPV**	9.6%	12.7%	14.0%
**NPV**	96.9%	98.2%	95.1%
**45+ years (835 women, 42 CIN3+)**			
**Positivity**	56.9%	50.1%	17.4%
**Sensitivity**	83.3%	97.6%	50.0%
**Specificity**	44.5%	52.2%	84.6%
**PPV**	7.4%	9.8%	14.7%
**NPV**	98.1%	99.8%	97.0%

ASC-US: Atypical Squamous Cells of Undetermined Significance; PPV: positive predictive value; NPV: negative predictive value; CIN3+: cervical intraepithelial neoplasia grade 3 or worse

© 2019 Wentzensen N et al. *JAMA Internal Medicine*.

eTable 2. Risk of CIN3+ and CIN2+ in Strata of Cytology, HPV16/18 Genotype, and Dual Stain Results

Stratum	All women in stratum	CIN3+ in stratum	Risk	95% Confidence Interval	CIN2+ in stratum	Risk	95% Confidence Interval
HSIL cytology; HPV16/18+; DS+	52	33	63.5%	49.0 - 76.4%	44	84.6%	71.9 - 93.1%
HSIL cytology; HPV16/18+; DS-	0	0	NE	-	0	NE	-
HSIL cytology; HPV16/18-; DS+	44	26	59.1%	43.2 - 73.7%	32	72.7%	57.2 - 85.0%
HSIL cytology; HPV16/18-; DS-	4	1	25.0%	0.6 - 80.6%	1	25.0%	0.6 - 80.6%
ASCH cytology; HPV16/18+; DS+	52	20	38.5%	25.3 - 53.0%	35	67.3%	52.9 - 79.7%
ASCH cytology; HPV16/18+; DS-	5	0	0.0%	0.0 - 52.2%	1	20.0%	0.5 - 71.6%
ASCH cytology; HPV16/18-; DS+	87	24	27.6%	18.5 - 38.2%	42	48.3%	37.4 - 59.2%
ASCH cytology; HPV16/18-; DS-	11	0	0.0%	0.0 - 28.5%	2	18.2%	2.3 - 51.8%
LSIL cytology; HPV16/18+; DS+	96	22	22.9%	14.9 - 32.6%	37	38.5%	28.8 - 49.0%
LSIL cytology; HPV16/18+; DS-	24	0	0.0%	0.0 - 14.2%	1	4.2%	0.1 - 21.1%
LSIL cytology; HPV16/18-; DS+	341	16	4.7%	2.7 - 7.5%	54	15.8%	12.1 - 20.2%
LSIL cytology; HPV16/18-; DS-	262	4	1.5%	0.4 - 3.9%	15	5.7%	3.2 - 9.3%
ASCUS cytology; HPV16/18+; DS+	101	23	22.8%	15.0 - 32.2%	39	38.6%	29.1 - 48.8%
ASCUS cytology; HPV16/18+; DS-	72	3	4.2%	0.9 - 11.7%	9	12.5%	5.9 - 22.4%
ASCUS cytology; HPV16/18-; DS+	361	20	5.5%	3.4 - 8.4%	54	15.0%	11.4 - 19.1%
ASCUS cytology; HPV16/18-; DS-	393	7	1.8%	0.7 - 3.6%	17	4.3%	2.5 - 6.8%
NILM cytology; HPV16/18+; DS+	114	14	12.3%	6.9 - 19.7%	23	20.2%	13.2 - 28.7%
NILM cytology; HPV16/18+; DS-	107	6	5.6%	2.1 - 11.8%	15	14.0%	8.1 - 22.1%
NILM cytology; HPV16/18-; DS+	362	11	3.1%	1.5 - 5.4%	31	8.6%	5.9 - 12.0%
NILM cytology; HPV16/18-; DS-	735	6	0.8%	0.3 – 1.8%	20	2.7%	1.7 - 4.2%

HPV16/18+: positive for either HPV16 or HPV18; DS: Dual stain cytology; NILM: negative for intraepithelial lesion or malignancy, ASC-US: Atypical Squamous Cells of Undetermined Significance, LSIL: Low Grade Squamous Intraepithelial Lesions; ASC-H: Atypical Squamous Cells, Cannot Rule Out High-Grade Squamous Intraepithelial Lesion; HSIL: High Grade Squamous Intraepithelial Lesion; CIN3+: cervical intraepithelial neoplasia grade 3 or worse, CIN2+: cervical intraepithelial neoplasia grade 2 or worse; NE, not estimable

© 2019 Wentzensen N et al. *JAMA Internal Medicine*.

eTable 3. HPV16/18 and p16/Ki-67 Positivity by Histology and Cytology Results in 2066 Women Without Known Preceding Abnormal Co-Test

**Cytology Result**	**Total N=2,066**		**No Biopsy N=588**		**Benign N=372**		**CIN1 N=783**		**CIN2 N=146**		**CIN3/ AIS N=130**		**Cancer N=1**		**Other N=46**	
	n	%	n	%	n	%	n	%	n	%	n	%	n	%	n	%
**Negative**	877	42%	550	94%	126	34%	138	18%	31	21%	20	15%	0	0%	12	26%
HPV16/18+	143	16%	86	16%	16	13%	17	12%	12	39%	10	50%	0	0%	2	17%
HR HPV12+	588	67%	367	67%	88	70%	101	73%	15	48%	9	45%	0	0%	8	67%
DS+	303	35%	173	32%	42	33%	50	36%	18	58%	15	75%	0	0%	5	42%
**ASC-US**	605	29%	21	4%	165	44%	333	43%	44	30%	21	16%	0	0%	21	46%
HPV16/18+	103	17%	3	14%	23	14%	48	14%	13	30%	12	57%	0	0%	4	19%
HR HPV12+	393	65%	13	62%	108	66%	225	68%	26	59%	7	33%	0	0%	14	67%
DS+	289	48%	9	43%	67	41%	158	48%	31	71%	18	86%	0	0%	6	29%
**LSIL**	432	21%	16	3%	70	19%	271	35%	39	27%	24	19%	0	0%	12	26%
HPV16/18+	72	17%	2	12.5%	6	9%	41	15%	10	26%	11	46%	0	0%	2	17%
HR HPV12+	286	66%	11	69%	52	74%	179	66%	25	64%	12	50%	0	0%	7	58%
DS+	254	59%	9	56%	40	57%	145	54%	30	77%	21	88%	0	0%	9	75%
**ASC-H**	93	5%	1	0%	8	2%	32	4%	25	17%	27	21%	0	0%	0	0%
HPV16/18+	31	33%	1	0%	2	25%	8	25%	9	36%	12	44%	0	0%	0	0%
HR HPV12+	50	54%	1	100%	4	50%	21	66%	13	52%	11	41%	0	0%	0	0%
DS+	82	88%	0	0%	6	75%	27	84%	22	88%	27	100%	0	0%	0	0%
**HSIL**	59	3%	0	0%	3	1%	9	1%	7	5%	38	29%	1	100%	1	2%
HPV16/18+	34	58%	0	0%	1	33%	4	44%	5	71%	22	58%	1	100%	1	100%
HR HPV12+	18	31%	0	0%	1	33%	3	33%	2	29%	12	32%	0	0%	0	0%
DS+	55	93%	0	0%	2	67%	7	78%	7	100%	37	97%	1	100%	1	100%
**Total**	2,066	100%	588	28%	372	18%	783	38%	146	7%	130	6%	1	0%	46	2%
HPV16/18+	383	19%	91	15%	48	13%	118	15%	49	34%	67	52%	1	100%	9	20%
HR HPV12+	1,335	65%	392	67%	253	68%	529	68%	81	56%	51	39%	0	0%	29	63%
DS+	983	48%	191	32%	157	42%	387	49%	108	74%	118	91%	1	100%	21	46%

HPV16/18+: positive for either HPV16 or HPV18; HR HPV12+: positive for at least one of the other high-risk HPV types; DS: Dual stain cytology; NILM: negative for intraepithelial lesion or malignancy, ASC-US: Atypical Squamous Cells of Undetermined Significance, LSIL: Low Grade Squamous Intraepithelial Lesions; ASC-H: Atypical Squamous Cells, Cannot Rule Out High-Grade Squamous Intraepithelial Lesion; HSIL: High Grade Squamous Intraepithelial Lesion, CIN: cervical intraepithelial neoplasia (grades 1-3), AIS, adenocarcinoma in situ

© 2019 Wentzensen N et al. *JAMA Internal Medicine*.

eTable 4. Performance of Cytology, Dual Stain, HPV16/18 and Combinations Among 2066 HPV-Positive Women Without Preceding Abnormal Co-Test to Detect CIN3+ (n=131) and CIN2+ (n=277)

	**Cytology + HPV16/18 (95% CI)**	**Dual stain + HPV16/18 (95% CI)**	**Cytology (95% CI)**	**Dual stain (95% CI)**
**Threshold**	Either ASC-US+ or HPV16 or HPV18 positive	1 dual stain positive cell or HPV16 or HPV18 positive	ASC-US+	1 dual stain positive cell
**Positivity**	1332/2066 (64.7%; 62.6-66.7%)	1113/2066 (54.0%[**]; 51.8-56.2%)	1189/2066 (57.8%[**]; 55.6-60.0%)	983/2066 (47.6%[**]; 45.4-50.0%)
**Detection of CIN3+ (n=131)**				
**Sensitivity**	121/131 (92.4%; 87.8-96.9%)	121/131 (92.4%; 87.8-96.9%)	111/131 (84.7%; 78.6-90.9%)[*]	119/131 (90.8%; 85.9-95.8%)
**Specificity**	724/1935 (37.2%; 35.3-39.6%)	943/1935 (48.7%; 46.5-51.0%)[**]	857/1935 (44.3%; 42.1-46.5%)[**]	1071/1935 (55.3%; 53.1-57.6%)[**]
**PPV**	121/1332 (9.1%; 7.5-10.6%)	121/1113 (10.9%; 9.0-12.7%)[**]	111/1189 (9.3%; 7.7-11.0%)	119/983 (12.1%; 10.1-14.1%)[**]
**NPV**	724/734 (98.6%; 97.8-99.5%)	943/953 (99.0%; 98.3-99.6%)	857/877 (97.7%; 96.7-98.7%)[*]	1071/1083 (98.9%; 98.3-99.5%)
**Detection of CIN2+ (n=277)**				
**Sensitivity**	248/277 (89.5%; 85.9-93.1%)	242/277 (87.4%; 83.5-91.3%)	226/277 (81.6%; 77.0-86.2%)[**]	227/277 (81.9%; 77.4-86.5%)[*]
**Specificity**	705/1789 (39.4%; 37.1-41.7%)	921/1789 (51.3%; 49.0-53.6%)[**]	826/1789 (46.2%; 43.9-48.5%)[**]	1033/1789 (57.7%; 55.4-60.0%)[**]
**PPV**	248/1332 (18.6%; 16.5-20.7%)	242/1113 (21.7%; 19.3-24.2%)[**]	226/1189 (19.0%; 16.8-21.2%)	227/983 (23.1%; 20.5-25.7%)[**]
**NPV**	705/734 (96.0%; 94.6-97.5%)	918/953 (96.3%; 95.1-97.5%)	826/877 (94.2%; 92.6-95.7%)[*]	1033/1083 (95.4%; 94.1-96.6%)

[*]p<0.05; [**]p<0.001 compared to cytology + HPV16/18 calculated using McNemar test for positivity and sensitivity and specificity and the Leisenring method for predictive values. ASC-US+: Atypical Squamous Cells of Undetermined Significance or more severe cytologic diagnosis, PPV: positive predictive value, NPV: negative predictive value, CIN3+: cervical intraepithelial neoplasia grade 3 or worse, CIN2+: cervical intraepithelial neoplasia grade 2 or worse

© 2019 Wentzensen N et al. *JAMA Internal Medicine*.

eTable 5. Risk of CIN3+ and CIN2+ in Strata of Cytology, HPV16/18 Genotype and Dual Stain Results in Women With Preceding Normal Co-Tests

Stratum	All women in stratum	CIN3+ in stratum	Risk	95% Confidence Interval	CIN2+ in stratum	Risk	95% Confidence Interval
HSIL cytology; HPV16/18+; DS+	34	23	67.7%	49.5 - 82.6%	28	82.4%	65.5 - 93.2%
HSIL cytology; HPV16/18+; DS-	0	0	NE	-	0	NE	
HSIL cytology; HPV16/18-; DS+	21	15	71.4%	47.8 - 88.7%	17	81.0%	58.1 - 94.6%
HSIL cytology; HPV16/18-; DS-	4	1	25.0%	4.6 - 69.9%	1	25.0%	4.6 - 69.9%
ASCH cytology; HPV16/18+; DS+	28	12	42.9%	24.5 - 62.8%	20	71.4%	51.3 - 86.8%
ASCH cytology; HPV16/18+; DS-	3	0	0.0%	0.0 - 70.8%	1	33.3%	0.8 - 90.6%
ASCH cytology; HPV16/18-; DS+	54	15	27.8%	16.5 - 41.6%	29	53.7%	39.6 - 67.4%
ASCH cytology; HPV16/18-; DS-	8	0	0.0%	0.0 - 36.9%	2	25.0%	7.2 – 59.1%
LSIL cytology; HPV16/18+; DS+	61	11	18.0%	9.4 - 30.0%	20	32.8%	21.3 - 46.0%
LSIL cytology; HPV16/18+; DS-	11	0	0.0%	0.0 - 28.5%	1	9.1%	0.2 - 4.3%
LSIL cytology; HPV16/18-; DS+	193	10	5.2%	2.5 - 9.3%	31	16.1%	11.2 - 22.0%
LSIL cytology; HPV16/18-; DS-	167	3	1.8%	0.4 - 5.2%	11	6.6%	3.3 - 11.5%
ASCUS cytology; HPV16/18+; DS+	59	11	18.6%	9.7 - 30.9%	20	33.9%	22.1 - 47.4%
ASCUS cytology; HPV16/18+; DS-	44	1	2.3%	0.1 - 12.0%	5	11.4%	3.8 - 24.6%
ASCUS cytology; HPV16/18-; DS+	230	7	3.0%	1.2 - 6.2%	29	12.6%	8.6 - 17.6%
ASCUS cytology; HPV16/18-; DS-	272	2	0.7%	0.1 - 2.6%	11	4.0%	2.0 - 7.1%
NILM cytology; HPV16/18+; DS+	71	9	12.7%	6.0 - 22.7%	14	19.7%	11.2 - 30.9%
NILM cytology; HPV16/18+; DS-	72	1	1.4%	0.0 - 7.5%	8	11.1%	5.7 - 20.4%
NILM cytology; HPV16/18-; DS+	232	6	2.6%	1.0 - 5.5%	19	8.2%	5.0 - 12.5%
NILM cytology; HPV16/18-; DS-	502	4	0.8%	0.2 - 2.0%	10	2.0%	1.0 - 3.6%

HPV16/18+: positive for either HPV16 or HPV18; DS: Dual stain cytology; NILM: negative for intraepithelial lesion or malignancy, ASC-US: Atypical Squamous Cells of Undetermined Significance, LSIL: Low Grade Squamous Intraepithelial Lesions; ASC-H: Atypical Squamous Cells, Cannot Rule Out High-Grade Squamous Intraepithelial Lesion; HSIL: High Grade Squamous Intraepithelial Lesion; CIN3+: cervical intraepithelial neoplasia grade 3 or worse, CIN2+: cervical intraepithelial neoplasia grade 2 or worse; NE, not estimable

© 2019 Wentzensen N et al. *JAMA Internal Medicine*.

eFigure 1. Screening and Triage Algorithms

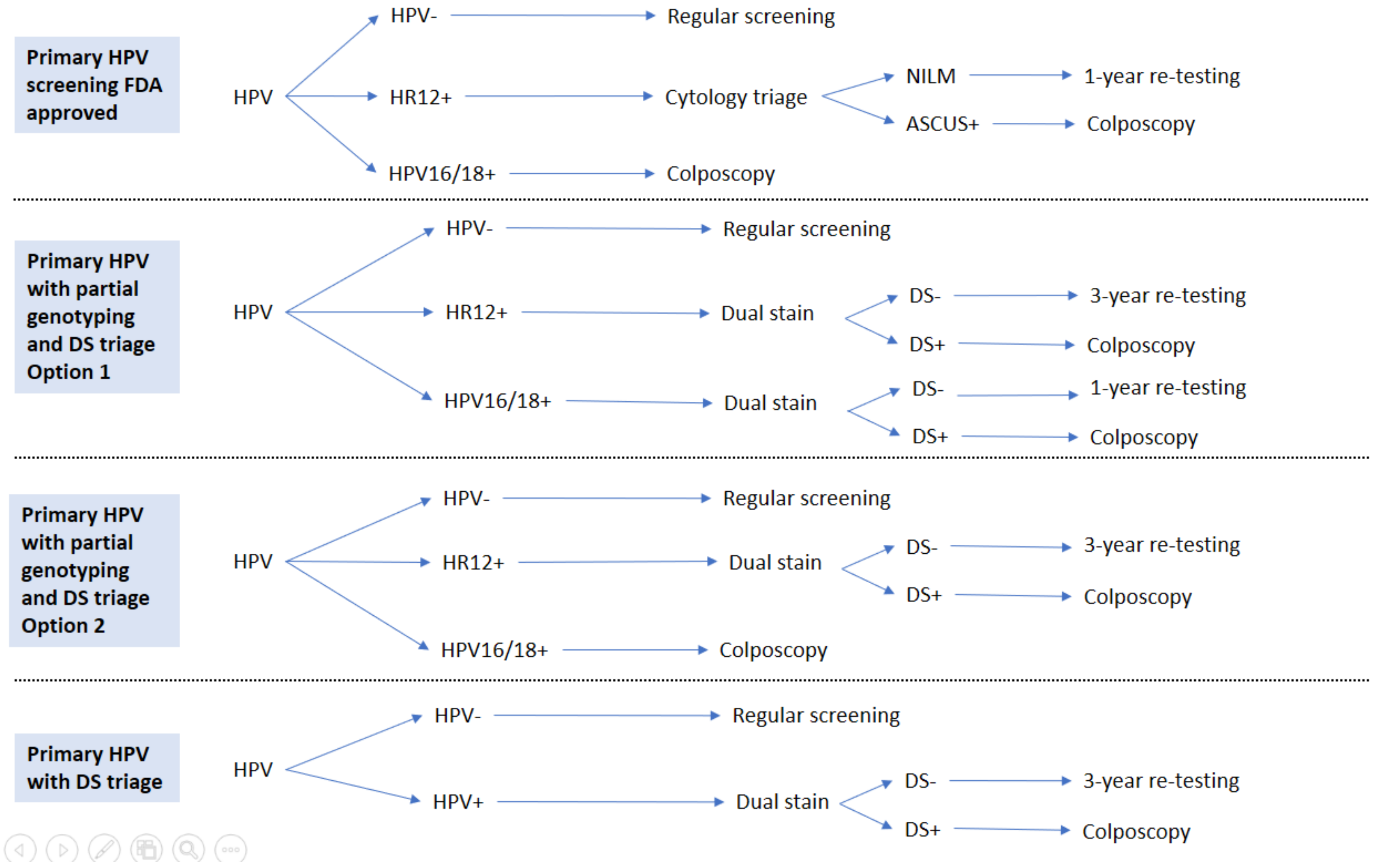

© 2019 Wentzensen N et al. *JAMA Internal Medicine*.

eFigure 2. Risk of CIN3+ in Strata of Cytology, Dual Stain, and HPV16/18 Among Women With Normal Preceding Co-Test

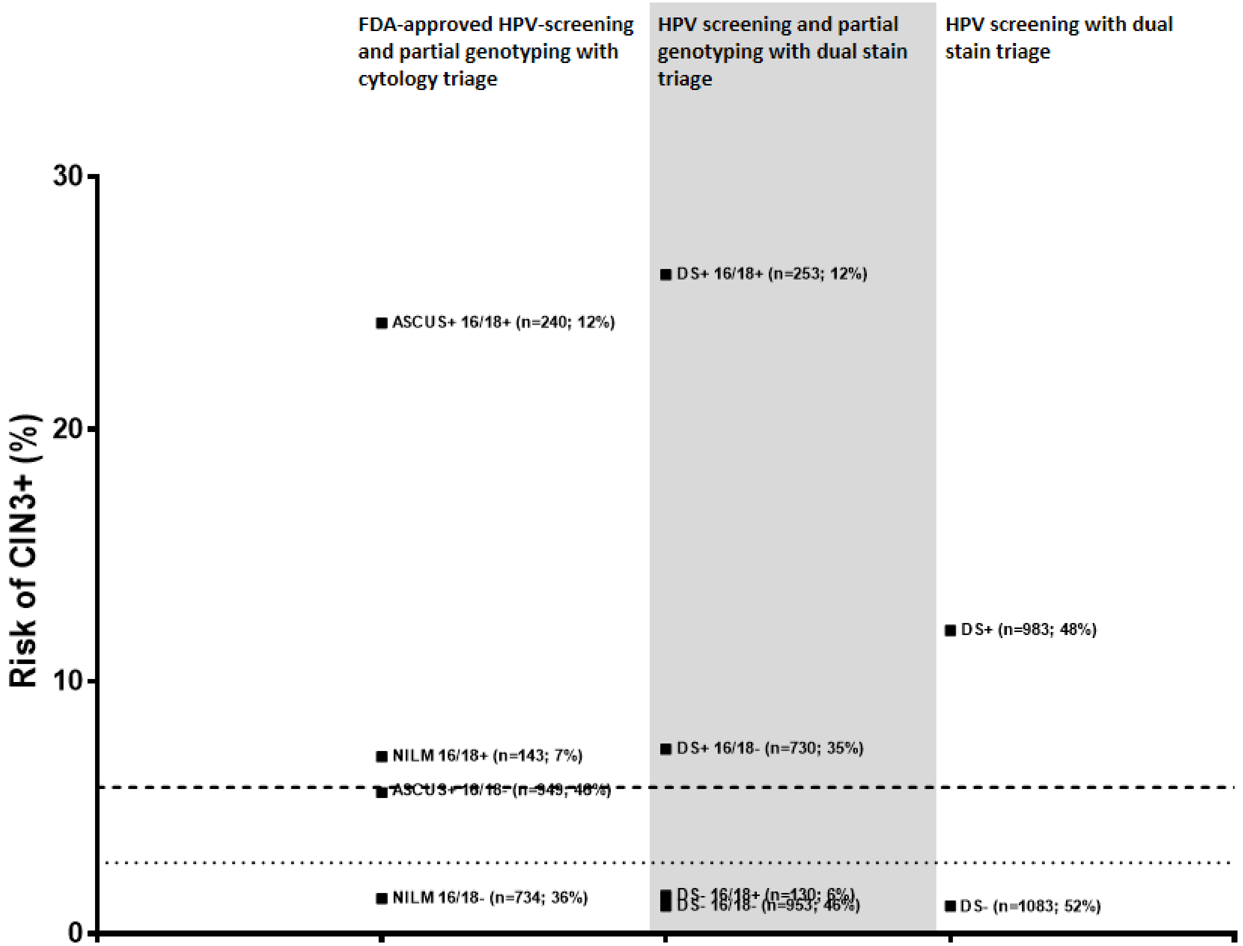

Dotted line: 1-year return threshold (HPV-positive NILM, 2.8%); dashed line: Colposcopy referral threshold (HPV-positive ASC-US, 5.7%). The number and corresponding percentage of women testing positive for each test result is shown in parentheses. Absolute risk of CIN3+ is plotted on the y-axis. Abbreviations: NILM: negative for intraepithelial lesion or malignancy, ASC-US+: Atypical Squamous Cells of Undetermined Significance or more severe cytologic diagnosis, 16/18+/-: HPV16/18 positive or HPV16/18 negative, respectively, DS+/-: dual stain positive or dual stain negative, respectively; CIN3+, cervical intraepithelial neoplasia grade 3 or more severe histologic diagnosis.

© 2019 Wentzensen N et al. *JAMA Internal Medicine*.
